# Resident fibroblasts in the kidney: a major driver of fibrosis and inflammation

**DOI:** 10.1186/s41232-017-0048-3

**Published:** 2017-08-07

**Authors:** Yuki Sato, Motoko Yanagita

**Affiliations:** 1 Medical Innovation Center, TMK project, Graduate School of Medicine, Kyoto University, Kyoto, Japan; 2 Department of Nephrology, Graduate School of Medicine, Kyoto University, Kyoto, Japan

**Keywords:** Fibroblast, Inflammation, Myofibroblast, Chronic kidney disease, Erythropoietin, Heterogeneity, Tertiary lymphoid tissue, CXCL13

## Abstract

**Background:**

Chronic kidney disease (CKD) is a leading cause of end stage renal disease (ESRD) and cardiovascular morbidity and mortality worldwide, resulting in a growing social and economic burden. The prevalence and burden of CKD is anticipated to further increase over the next decades as a result of aging.

**Main body of abstract:**

In the pathogenesis of CKD, irrespective of the etiology, resident fibroblasts are key players and have been demonstrated to play crucial roles for disease initiation and progression. In response to injury, resident fibroblasts transdifferentiate into myofibroblasts that express alpha smooth muscle actin (αSMA) and have an increased capacity to produce large amounts of extracellular matrix (ECM) proteins, leading to renal fibrosis. In addition to this fundamental role of fibroblasts as drivers for renal fibrosis, growing amounts of evidence have shown that resident fibroblasts are also actively involved in initiating and promoting inflammation during kidney injury. During the myofibroblastic transition described above, resident fibroblasts activate NF-κB signaling and produce pro-inflammatory cytokines and chemokines, promoting inflammation. Furthermore, under aging milieu, resident fibroblasts transdifferentiate into several distinct phenotypic fibroblasts, including CXCL13/CCL19-producing fibroblasts, retinoic acid-producing fibroblasts, and follicular dendritic cells, in response to injury and orchestrate tertiary lymphoid tissue (TLT) formation, which results in uncontrolled aberrant inflammation and retards tissue repair. Anti-inflammatory agents can improve myofibroblastic transdifferentiation and abolish TLT formation, suggesting that targeting these inflammatory fibroblasts can potentially ameliorate kidney disease.

**Short conclusion:**

Beyond its conventional role as an executor of fibrosis, resident fibroblasts display more pro-inflammatory phenotypes and contribute actively to driving inflammation during kidney injury.

## Background

Fibroblasts reside in virtually all tissues in our body and provide three-dimensional architecture and mechanical strength to the tissues. Emerging evidence suggests that they also have tissue-specific physiologic functions and participate actively in pathogenesis during injury. In the kidney, resident fibroblasts produce erythropoietin (EPO) in response to hypoxic insults to maintain homeostasis under physiologic condition, whereas, under pathologic conditions, resident fibroblasts transdifferentiate into myofibroblasts, which execute renal fibrosis by producing large amounts of extracellular matrix proteins, at the cost of EPO production [1, 2]. Recently, the role and phenotype of resident fibroblasts in the kidney during injury have been demonstrated to be more diverse and crucial for disease initiation and progression. Under the aging milieu, for example, resident fibroblasts further gain a variety of distinct phenotypes in response to injury and orchestrate tertiary lymphoid tissue formation, which results in uncontrolled inflammation and retards tissue repair [3]. In this review, we provide the current state of knowledge of the renal fibroblasts as a driver of fibrosis and inflammation, and consider a novel therapeutic strategy to treat patients with kidney disease.

### Resident fibroblasts as sentinels in the kidney

The kidney plays a central role in body fluid homeostasis and metabolic waste elimination. Each human kidney is composed of about 1 million nephrons, which are functional units of the kidney that comprise the glomerulus and the tubules. The glomerulus is a capillary loop that is specialized for plasma filtration. The glomeruli receive blood supply from the renal artery, and the glomerular filtrate subsequently travels through renal tubules, where metabolic exchange, and reabsorption and secretion occur. Resident fibroblasts are spindle-shaped mesenchymal cells that reside in the renal interstitium [4], which is the extracellular compartment between tubules and peritubular capillaries [5]. Several pathological studies have shown that the magnitude of renal impairment correlates better with interstitial changes than the glomerular changes in most forms of chronic kidney disease (CKD), suggesting that renal function is critically dependent on the environment in this compartment.

The kidney interstitium contains two cellular components: resident fibroblasts and resident renal mononuclear phagocytes (rMoPh) [6, 7]. Although these two types of cells reside in virtually all tissues, they are versatile cell types with strong organ-specific modifications. The kidney is continually exposed to various kinds of endogenous and exogenous substances, which must be monitored and possibly eliminated, and most of the reabsorbed substances have to traverse the interstitium before entering the capillaries. Fibroblasts and rMoPh are strategically positioned at the interstitium to sense these circulating substances and environmental changes [5]. Indeed, with the progression of kidney disease, many kinds of uremic toxins have accumulated in the body and have various effects on these renal cells. For instance, indoxyl sulfate (IS), a typical uremic toxin derived from indole, suppresses EPO production in resident fibroblasts in the kidney [8], which may partly explain the relative deficiency of EPO production in CKD patients described in the next chapter.

The readiness to respond to diverse environmental cues has been well described for rMoPh, and these cells have been considered to be sentinels in the kidney [6]. However, it has recently been demonstrated that fibroblasts also express most immune receptors, including pattern recognition receptors such as Toll-like receptors (TLRs), and that they are also highly sensitive to local tissue injury. Leaf et al. demonstrated that, although various types of cell are likely to become activated through TLRs, fibroblasts respond to damage-associated molecular patterns (DAMPs) more sensitively than other cell types including epithelial cells, endothelial cells, and even monocyte-derived macrophage in the context of sterile inflammation, and they produce higher amount of pro-inflammatory cytokine, participating actively in the initiation of renal inflammation [9]. Macrophages, on the other hand, have a higher sensitivity to pathogen ligands, suggesting that these two cell types may collaborate together and serve as a sophisticated network that senses both intrinsic and extrinsic substances under physiologic and pathologic conditions.

Pericytes, which are defined as mesenchymal cells wrapping around the microvessels, also reside in interstitium and are positive for CD73 and PDGFRβ, both of which are also utilized as markers for resident fibroblasts [10]. Overlapping definitions of resident fibroblasts and pericytes have generated confusion and controversy, although it is becoming increasingly clear that they are overlapping populations in the kidney [11]. Recent studies of the lineage relationships demonstrated that almost all fibroblasts in the renal cortex and outer medulla, including EPO-producing cells, are derived from cells that are lineage-labeled with *myelin protein zero (P0)-Cre* [12], which labels migrating neural crest cells and neural crest-derived Schwann cells [13], whereas almost all pericytes are derived from *Foxd1-Cre* lineage-labeled stromal cells [14]. *P0-Cre* lineage-labeled cells transiently express FoxD1 during development, whereas FoxD1 is expressed in the migrating neural crest, indicating that these two populations are overlapping [11].

### Role of resident fibroblasts during kidney injury

Fibrosis is a common pathologic feature in CKD patients, and myofibroblasts are major drivers of fibrosis. Myofibroblasts are not present under physiologic conditions, but emerge de novo in injured tissues. The origin of myofibroblasts has been controversial for a long time, and various precursor cells of myofibroblasts in fibrotic kidneys have been reported [1]. Over the last 5 years, comprehensive cell fate mapping experiments using various Cre mouse lines have been conducted by several groups and the origin of myofibroblasts has been reconsidered. We also demonstrated that *P0-Cre* lineage-labeled resident fibroblasts transdifferentiate αSMA-positive myofibroblasts in response to kidney injury [12]. Together with the results from other groups, it is currently believed that resident fibroblasts or pericytes seem to be the most important precursor of myofibroblasts, which is consistent with recent studies in liver [15], lung [16, 17], and skin fibrosis [18, 19], all of which concluded that myofibroblasts are derived from resident fibroblasts. Interestingly, Kramman et al. recently identified the myofibroblast progenitor, which represents a small fraction of renal pericytes in the healthy kidney, and they were lineage-labeled with Gli1 [20]. Gli1-positive pericytes fulfill the criteria of mesenchymal stem cells, having tri-lineage differentiation potential and colony-forming capability in vitro. The blood vessel wall was shown to be a niche for mesenchymal stem cells in multiple human organs including the skeletal muscle, pancreas, adipose tissue, placenta, and kidney [21, 22].

In CKD patients, fibrosis progresses for decades. This clinical course suggests that epigenetic changes, which can persist long after the removal of initial trigger, have been involved and played an important role in this process. A recent genome-wide methylation scan of fibroblasts in the kidney identified epigenetic silencing of RASAL1, a suppressor of the Ras oncoprotein, as the cause of spontaneous proliferation of fibroblasts [23], providing a new molecular explanation for a sustained activation of fibroblasts in the injured kidneys.

In parallel with renal fibrosis, as the severity of kidney disease progresses, renal anemia increases in prevalence. Renal anemia is driven predominantly by a relative deficiency in the production of EPO, a principal regulatory hormone of red blood cell production [2], which is produced by renal resident fibroblasts in response to hypoxia [24]. We have previously demonstrated that, during kidney injury, EPO-producing cells transdifferentiate into myofibroblasts, same as other resident fibroblasts in the kidney, at the cost of EPO production [12]. EPO production is mainly regulated by hypoxia-inducible factors (HIFs) in healthy kidney. However, in injured kidneys, despite their hypoxic conditions, most of the HIF target gene expression is insufficient to counteract hypoxia [25]. Notably, we also showed that these transdifferentiated myofibroblasts regain their EPO production ability after the induction of severe anemia or the administration of neuroprotective agents such as neurotrophin and selective estrogen receptor modulator (SERM) [12]. These results indicate that resident fibroblasts possess functional plasticity and myofibroblasts still have the potential to produce EPO in response to hypoxic insults, which is consistent with epidemiological evidence indicating the presence of hypoxia-driven EPO regulation even in the patients with ESRD who require hemodialysis [26].

What triggers this phenotypic change in fibroblast in the kidney? In the previous study, we have demonstrated that proximal tubule injury alone can drive this phenotypic change and lead to renal fibrosis and deficiency in EPO production [27]. This phenomenon has been supported by the evidences from other groups, which demonstrate that TGF-β derived from injured tubules promotes the transdifferentiation from fibroblast into myofibroblast [28]. In addition to this, the pathways regulating this phenotypic change, including PDGFR pathway and hedgehog pathway, have been already identified and considered as targets of novel therapeutic approaches [11]. Interestingly, during this phenotypic transition, fibroblasts also become both extracellular matrix (ECM)-producing cells and inflammatory effector cells [29, 30]. In response to injury, renal fibroblasts have been shown to activate NF-κB signaling, which leads to the production of pro-inflammatory cytokines and chemokines. The pro-inflammatory cytokines such as IL-1 and TNFα activate GATA-2 and NF-κB, both of which inhibit EPO transcription by binding the EPO promoter [29, 31], leading to relative EPO deficiency in CKD patients. Together with the findings that anti-inflammatory agents such as dexamethasone can restore the myofibroblast phenotype [12, 29], the inflammatory phenotypes of fibroblasts can be a promising therapeutic target and require more characterization in future studies.

In addition, various types of cells in the kidney, including fibroblasts, produce prostaglandins (PGs), which have also been recognized as a mediator of inflammatory responses [32]. Growing amounts of evidence have shown that PGs are involved in tissue fibrosis and inflammation. PGE2 is the most abundant PG in the kidney, and it plays a suppressive role in renal fibrosis via its receptor EP4 [33], although specific EP4 depletion in podocytes, which are a critical component of the filtration barrier in the glomerulus, results in milder glomerular injury [34]. These potential confounding features suggest that the PG cellular source and function are highly variable depending on the conditions and the cell type. PG signaling is considered to be a promising therapeutic target because PGs have been shown to amplify cytokine signaling and induce chemokine expression in other organs [32].

### Heterogeneity of resident fibroblasts involved in tertiary lymphoid tissue formation in aged injured kidney

**Fig. 1:**
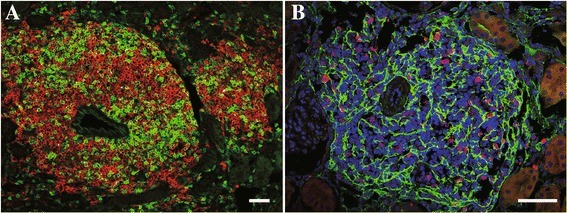
Tertiary lymphoid tissues in aged injured mouse kidney. Tertiary lymphoid tissues are mainly composed of T cells and B cells, some of which are proliferating. p75NTR-positive fibroblasts extend their projections and form a structural backbone within TLTs. **a** *green*: CD3ε, *red*: B220. **b** *green*: p75NTR, *red*: Ki67. *Scale bar* (**a**, **b**) 50 μm

Several phenotypically novel heterogeneous fibroblasts in the injured kidney have been identified recently, and they were characterized in both the rodent and human kidneys, which are involved in tertiary lymphoid tissue (TLT) formation [3]. TLTs are inducible ectopic lymphoid tissues that are composed of a hematopoietic compartment, which comprises mostly T cells and B cells, and stromal components, which include fibroblasts in particular (Fig. 1) [35]. TLTs can propagate local antigen-specific immune responses within non-lymphoid tissues, although their roles are context dependent and can be either beneficial or detrimental [36]. In chronic inflammatory disorders, for instance, TLTs are generally considered to be perpetuators of aberrant immune responses and detrimental to the host [37], whereas, during infections, TLTs generate robust immune responses to pathogens and play protective roles for the host [38]. Besides anatomical and functional similarities, TLT and secondary lymphoid organs, such as lymph nodes, both depend on related mechanisms and molecules for their development [39, 40]. In lymph nodes, homeostatic chemokines, including CXCL13, CCL19, and CCL21, play essential roles in their development, maturation, and homeostasis [39, 40]. The homeostatic chemokine is a powerful driving force for recruiting lymphocytes and is also sufficient to drive TLTs in non-lymphoid tissue, as transgenic expression of homeostatic chemokines in non-lymphoid organs induce the development of functional TLTs [41, 42].

**Fig. 2:**
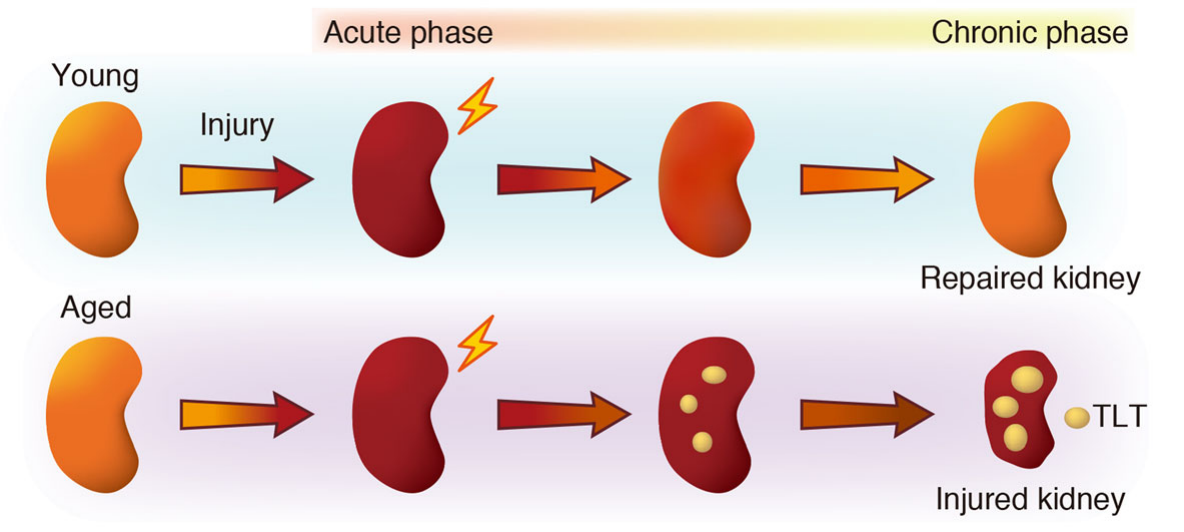
The distinct injury response in young and aged mice. Aged mice, but not young mice, developed multiple tertiary lymphoid tissues (TLTs) in the kidney after acute kidney injury (AKI). TLTs sustain and amplify inflammation and retard regeneration, resulting a poor renal outcome in aged mice

Recent studies have increasingly highlighted the potential roles for TLTs in regulating local immune responses in various pathological conditions. We showed that aged mice, but not young mice, developed multiple TLTs in the kidney after acute kidney injury (AKI) (Fig. 2). This unique response program in aged injured kidneys might explain why aged kidneys fail to repair themselves after kidney injury and progress to ESRD [43], since aberrant chronic inflammation hinders normal tissue repair and results in worse remodeling and dysfunction [44, 45]. Administration of anti-CD4 monoclonal antibody and dexamethasone abolished TLT formation and improved renal outcomes. Thus, the molecular mechanisms that govern the development and maintenance of TLT identity are of great interest, having implications for the prevention of TLT formation and the subsequent aberrant inflammation [46].

**Fig. 3:**
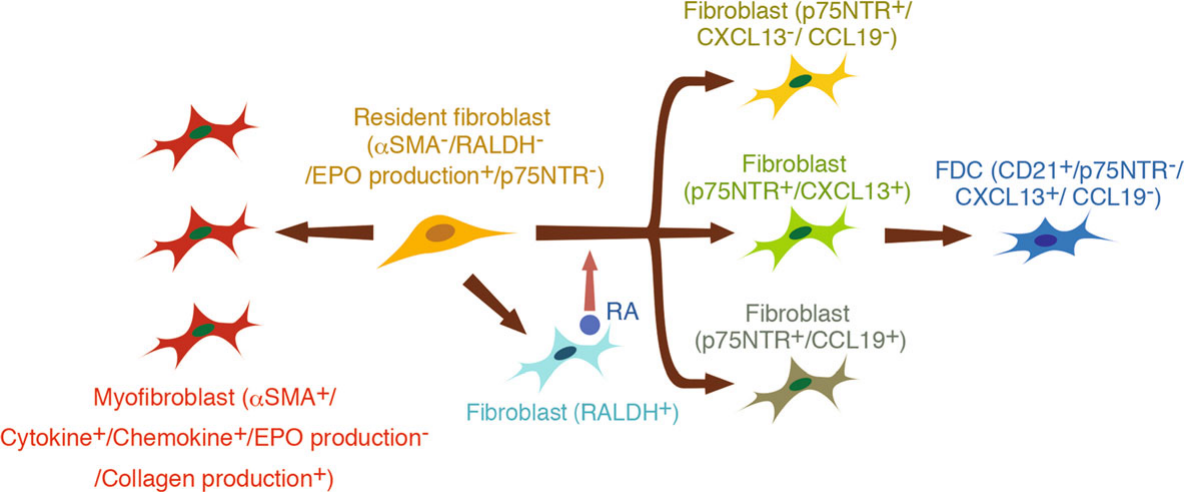
Fibroblasts have two jobs during CKD: fibrosis and inflammation. Resident fibroblasts critically contribute to fibrosis and the persistence of inflammation in the injured kidney [3]. Upon injury, resident fibroblasts transdifferentiate into myofibroblasts, which produce a large amount of ECM protein and pro-inflammatory cytokines/chemokines, at the cost of EPO production. Moreover, in the aging milieu, resident fibroblasts also transdifferentiate into several distinct phenotypic fibroblasts, which orchestrate TLT formation. In response to injury, resident fibroblasts differentiate into RALDH-positive fibroblasts, which induce transdifferentiation of other fibroblasts into p75NTR-positive fibroblasts with three phenotypes, which include CXCL13- and CCL19-producing fibroblasts. In the later phase of TLT formation, some of these p75NTR-positive fibroblasts lose this expression and mature into CD21/CXCL13-positive FDCs

In aged injured kidneys, resident fibroblasts transdifferentiate into myofibroblasts and also into several distinct phenotypic fibroblasts, which are involved in TLT formation (Fig. 3). After kidney injury, some resident fibroblasts acquire the ability to produce retinoic acid, which induces the neural crest marker p75NTR. Some p75NTR-positive fibroblasts in aged injured kidneys produce CXCL13 and CCL19, resulting in TLT formation [3]. Additionally, in the later phase of TLT formation, some of the p75NTR-positive fibroblasts appear to lose their p75NTR expression and mature into follicular dendritic cells (FDCs). FDCs express high amounts of CD21, complement receptors-2, and CXCL13, resulting in forming B cell areas and supporting germinal center response [47]. Lineage tracing demonstrated that *P0-Cre* lineage-labeled resident fibroblasts diversified into fibroblasts with these several distinct phenotypes essential for TLT formation (Fig. 3). These findings in renal TLT are consistent with the results of lineage-tracing studies in stromal cells in secondary lymphoid organs [48, 49], indicating that FDCs in the spleen are lineage-labeled with *PDGFRβ-Cre*, whereas FDCs in lymph nodes are lineage-labeled with *Wnt1-Cre*, which is another Cre line which labels neural crest-derived cells. Collectively, our results confirm and extend the concept that resident fibroblasts in the kidney exhibit striking plasticity and functional diversity depending on their residing microenvironments. One important remaining question to be addressed is “why the renal environment is prone to TLT formation with aging.” One possibility is the contribution of hematopoietic cell aging, especially CD4 positive T cells, because depletion of CD4 positive cells abolished TLTs [3]. Because of thymic involution, T cells undergo a global phenotype shift from naïve to memory T cells with aging, and unique age-dependent memory CD4 positive T cell subpopulation has been identified [50]. Another possibility is the contribution of aging in stromal cells, especially resident fibroblasts in the kidney. Further studies will be required to determine which cell aging is critical for the TLT formation.

The developmental mechanism of age-dependent TLTs in the kidney and inducible bronchus-associated lymphoid tissue (iBALT) in the lung is similar in that both TLTs are driven by CXCL13 and CCL19 [3, 51]. Although lymphoid tissue is normally absent in the lung, iBALT develops following to various kinds of infection and inflammatory diseases [52]. iBALT has separated T and B cell areas, some of which contain FDCs and germinal centers, and generate immune responses. Although various types of cells, such as monocyte-lineage cells and fibroblasts, have been reported as to be a source of CXCL13 in the lung, bone marrow chimera experiments have demonstrated that the majority of CXCL13 producing cells are non-hematopoietic cells in iBALT [53]. Rangel et al. have showed that wild type mice reconstituted with CXCL13-deficient bone marrow exhibited nearly identical lung expression of CXCL13 compared with wild type controls [53]. Furthermore, the same group has also demonstrated that interleukin-17 produced by CD4-positive T cells triggers the expression of CXCL13 and CCL19, but not CCL21, in pulmonary fibroblasts, which results in iBALT formation [51]. Altogether, these results suggested that resident fibroblasts have the potential to become homeostatic chemokine-producing cells in various organs. Though monocyte lineage cells have also been reported as CXCL13-producing cells in murine lupus models in the kidney [54, 55], the expression of CXCL13 in fibroblasts was not examined in these studies, and the relative contribution of hematopoietic cells and non-hematopoietic cells to overall CXCL13 expression in the kidney has yet to be determined in this model. Further studies are required to determine the main cellular source of renal CXCL13 in this model.

It is difficult to determine whether TLT is beneficial, harmful, or neutral for the host. This is partly because it is technically challenging to deplete TLTs specifically at any time without affecting the immune system systemically. Another way to determine whether TLTs play pathogenic roles is to determine whether TLTs produce autoantibodies. Given that TLTs lead to the production of tissue-specific autoantibodies, targeting TLT formation could be beneficial. Indeed, this idea has been already tested. Lehmann-Horn et al. demonstrated that in experimental autoimmune encephalomyelitis (EAE), autoantibodies with modified affinity for myelin self-antigens are generated within TLT in the meninges of central nerve system [56], suggesting the pathological roles of TLT in this context. In addition to the conventional roles of TLT as an amplifier of inflammation, recent studies have shown that, in some pathological conditions, TLTs can function as niches for tumor progenitor cells [57] and pathogenic memory T cells [58], which might represent new therapeutic targets for cancer and chronic inflammatory diseases. The role of TLTs may be variable and be influenced by the stage of the disease, site of formation, and various environmental factors, all of which determine the impact of TLTs on disease progression. Further studies are required to determine the precise roles of TLTs in various pathologic conditions.

## Conclusions

Dysfunction of resident fibroblasts leads to a series of clinically relevant pathological conditions that are common in CKD, indicating their importance in maintaining homeostasis under normal conditions. Beyond its conventional role as an executor of fibrosis, resident fibroblasts display more pro-inflammatory phenotypes and contribute actively to driving inflammation during kidney injury (Fig. 3), and intervention with anti-inflammatory agents has the potential to ameliorate kidney injury. Further studies are required to create novel therapeutic approaches, which may emerge as a consequence of a better understanding of the behavior of fibroblast under physiologic and pathologic conditions.

## Abbreviations

AKI: Acute kidney injury
CKD: Chronic kidney disease
DAMPs: Damage-associated molecular patterns
ECM: Extracellular matrix
EPO: Erythropoietin
ESRD: End stage renal disease
FDC: Follicular dendritic cell
iBALT: Inducible bronchus-associated lymphoid tissue
P0: Myelin protein zero
PG: Prostaglandin
rMoPh: Resident renal mononuclear phagocytes
TLR: Toll-like receptor
TLT: Tertiary lymphoid tissue
αSMA: Alpha smooth muscle actin
